# Preparation and Scintillating Properties of Sol-Gel Eu^3+^, Tb^3+^ Co-Doped Lu_2_O_3_ Nanopowders

**DOI:** 10.3390/ijms12096240

**Published:** 2011-09-23

**Authors:** Ángel de Jesús Morales Ramírez, Antonieta García Murillo, Felipe de Jesús Carrillo Romo, Margarita García Hernández, Joel Moreno Palmerin, Rosario Ruiz Guerrero

**Affiliations:** 1 Instituto Politécnico Nacional, CIITEC IPN, Cerrada de Cecati S/N. Col. Santa Catarina, Azcapotzalco Mexico D.F. C.P. 02250, Mexico; E-Mails: amoralesra@ipn.mx (Á.J.M.R.); fcarrillo@ipn.mx (F.J.C.R.); margarciah@ipn.mx (M.G.H.); maruizg@ipn.mx (R.R.G.); 2 CINVESTAV Querétaro, Libramiento Norponiente #2000, Fracc. Real de Juriquilla, C.P. 76230, Querétaro, Mexico; E-Mail: jmoreno@qro.cinvestav.mx

**Keywords:** sol-gel, nanopowders, Lu_2_O_3_:Eu^3+^-Tb^3+^, scintillation properties

## Abstract

Nanocrystalline Eu^3+^, Tb^3+^ co-doped Lu_2_O_3_ powders with a maximum size of 25.5 nm were prepared by the sol-gel process, using lutetium, europium and terbium nitrates as precursors, and ethanol as a solvent. Differential thermal analysis (DTA) and infrared spectroscopy (IR) were used to study the chemical changes during the xerogel annealing. After the sol evaporation at 100 °C, the formed gel was annealed from 300 to 900 °C for 30 min under a rich O_2_ atmosphere, and the yielded product was analyzed by X-ray diffraction (XRD) to characterize the microstructural behavior and confirm the crystalline structure. The results showed that Lu_2_O_3_ nanopowders start to crystallize at 400 °C and that the crystallite size increases along with the annealing temperature. A transmission electron microscopy (TEM) study of samples annealed at 700 and 900 °C was carried out in order to analyze the microstructure, as well as the size, of crystallites. Finally, in regard to scintillating properties, Eu^3+^ dopant (5 mol%), Tb^3+^ codoped Lu_2_O_3_ exhibited a typical red emission at 611 nm (D_°_→^7^F_2_), furthermore, the effect of Tb^3+^ molar content (0.01, 0.015 and 0.02% mol) on the Eu^3+^ radioluminiscence was analyzed and it was found that the higher emission intensity corresponds to the lower Tb^3+^ content.

## 1. Introduction

Since Lu_2_O_3_:Eu^3+^ first attracted attention as a potential X-ray phosphor [1], many efforts have been conducted in the last few decades to process it, due to a growing need for new materials to be employed in high-resolution X-ray imaging systems, including high-definition X-ray radiographers, positron emission tomography (PET) scanners as well as many others industrial measuring systems [2–5]. What makes lutetia attractive for such applications is its high efficiency in absorbing any kind of ionizing radiation [6]. Indeed, the combination of its high density (9.42 gcm^−3^) and its high atomic number of Lu (Z = 71) [7], along with its good absorption in the diagnostic medical energy range (15–150 keV) makes it a very interesting materials for the mentioned applications [8]. Furthermore, it is a convenient host lattice for activators forming scintillating materials [9], especially for Eu^3+^ and Tb^3+^, due to the fact that its band gap is large enough to accommodate the energy levels of the ions [10]. On the other hand, lutetia systems doped with Eu^3+^ are expected to replace the typical CsI:Tl scintillators in digital imaging, since, along with their stated properties, they present a reddish emission wavelength (~611 nm) that matches the spectral sensitivity of CCD detectors [11,12], and, moreover, CsI:Tl suffer from radiation damage at high doses, probably due to its low density (4.51 gcm^−3^) and is slightly hygroscopic [13,14]. Another common material, the bismuth orthogermanate (BGO), presents a low density (7.13 gcm^−3^) and emits less than 10 photons/keV [15], compared to 30 photons/keV for Lu_2_O_3_:Eu^3+^ [16]. Finally, the well known Gd_2_O_2_S:Tb^3+^ (GOS), tends to be replaced in scintillating devices due to its chemical instability and sensitivity to moisture [17,18]. Furthermore, it has been demonstrated that Lu_2_O_3_:Eu^3+^ presents a light yield similar to that of GOS [19] and that a scintillating screen made of this material can produce images of better quality than those obtained by the standard GOS [20]. On the other hand, it has been noted that different methods of synthesis lead to particles with diverse morphologies and specific surface areas, and, therefore, different luminescent properties [21,22]. Because it is essential for scintillating applications to yield ultrafine, mono-sized, low-aggregated and spherical powders [23], many soft chemical routes have been used to produce lutetia nanopowders, such as co-precipitation [24–26], solvothermal reaction [27–31], molten salts synthesis [32], and the combustion method [33–37]. Oxalic acid and urea have been used extensively in some of these methods, but these present some drawbacks for rare earth compounds, particularly due to the fact that rare earth oxalates produce particles that grow rapidly, along with severe agglomeration, whereas urea limits the yield from the homogeneous precipitation process [38]. Therefore, the sol-gel process, due to its unique advantages, like high chemical homogeneity, the possibility of achieving several compositions by simples changes in the process, and the ability to vary the nature and concentration of doping ions [39], it is ideal to produce rare earth doped Lu_2_O_3_ nanopowders [40–43]. Finally, in order to improve the light yield of these materials, the Tb^3+^ ion has been proposed for co-doping Lu_2_O_3_:Eu^3+^ [44]. In Y_2_O_3_:Eu^3+^,Tb^3+^, it has been found that there is an increment of the Eu^3+^ luminescence, due to an energy transfer from Tb^+3+^ to Eu^3+^ ions [45,46]; furthermore in previous work [47], it has been determined that, in Gd_2_O_3_:Eu^3+^ nanopowders, the radioluminescence emission is enhanced by the incorporation of Tb^3+^ ions.

The aim of the current work is to synthesize for first time Eu^3+^,Tb^3+^ co-doped Lu_2_O_3_ scintillating nanopowders by a simple sol-gel process, starting with nitrates as precursors and without the use of urea or oxalic acid, and instead, diethyleneglycol (DEG) C_4_H_10_O_3_ as polymerization agent. It was studied the chemical changes during the sol gel process of the nanopowders by means of a Thermogravimetric (TGA), differential thermal analyses (DTA) and infrared spectroscopy (IR). The structural evolution during the annealing process was analyzed by X-ray diffraction (XRD), the morphology by means of TEM observations, and their scintillating properties under X-ray (30 keV) radiation as a function of the heat treatment temperature and the Tb^3+^ content.

## 2. Results and Discussion

### 2.1. Thermal Analysis

**Figure 1:**
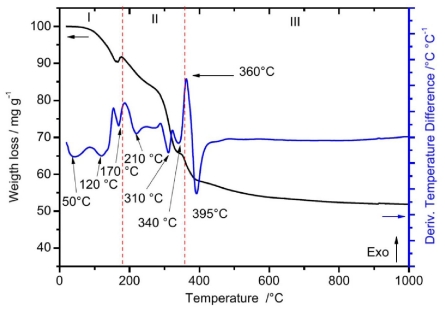
Differential Thermal Gravimetry (DTG) and differential thermal analyses (DTA) profiles of Lu_2_O_3_: 5 mol % Eu^3+^, 0.01 mol % Tb^3+^ xerogel powders.

Figure 1 shows the TGA-DTA curves of the as-prepared Lu_2_O_3_ xerogel 5 mol % Eu^3+^, 0.01 mol % Tb^3+^. As observed, the TGA curve shows three stages of weight loss. The first stage (I), from 100–175 °C, shows a weight loss of 9% and two endothermic events, at 120 and 170 °C. The first event can be attributed to the release of water of hydration and OH, and the second one to the decomposition of the organic matter, this event suggest the formation of lutetium hydrated species or even carbonate species to form precipitates with Lu^3+^ ions [48]. The second stage (II), from 175–360 °C, corresponding to a weight loss of 25%, involves two types of endothermic events, occurring at 210, 310 and 340°C. The first can be ascribed to the evaporation of DEG (b. p. 240 °C), whereas the last two correspond to the elimination of carbonyl groups. The last stage (III) exhibits a weight loss of 14% and includes two major events: a strong exothermic peak at 360 °C, which can be associated with the crystallization process of the ceramic sample into the cubic phase, and a strong endothermic peak at 390 °C, which was related to the pyrolisis of the remnant’s carbon groups.

### 2.2. Infrared Analysis

**Figure 2:**
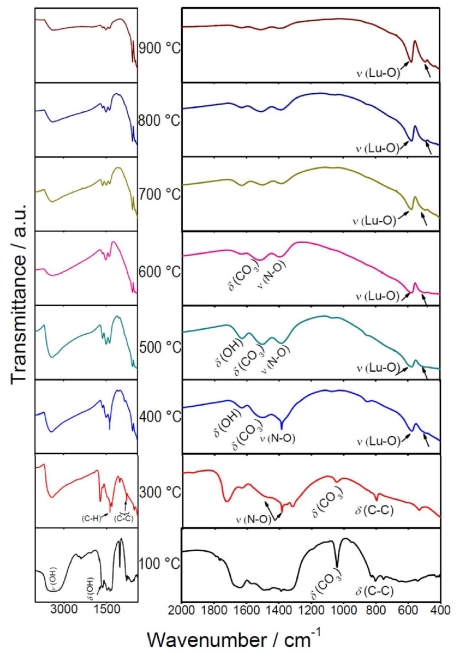
Infrared spectra of Lu_2_O_3_: 5 mol% Eu^3+^, 0.01 mol% Tb^3+^ powders at different annealing temperatures.

The Fourier transformed infrared spectra of Lu_2_O_3_: Eu^3+^ 5 mol %, Tb^3+^ 0.01 mol% are depicted in Figure 2. This study was carried out in the range 4000–400 cm^−1^ on the dried precursor sol, thermally treated at different temperatures, in order to determine the evolution of the decomposed products of the xerogel powders calcined up to the crystallization process. For the xerogel at 100 °C, bands observed at 3400 cm^−1^ (ν), 1650 cm^−1^ (δ) and 750 cm^−1^ (δ) can be ascribed to O-H stretching (ν) and deformation (δ) vibrations, due the presence of water and alcohol groups. Since the heating at 400 °C was not enough to remove these species, we have to conclude that they were structurally built into the host and not merely adsorbed on the surface. After a 600 °C heat treatment, these O-H vibrations were less intense than those observed at lower temperatures, exerting an influence on the powder’s microstructure, as demonstrated by XRD analysis. While the heating of the powders at much higher temperatures (necessarily to stimulate the growth of the crystallites and their partial sintering), the vibrations related with the O-H impurities [49] are almost absent, *i.e*., these vibrations are nearly missing at 900 °C. The absorption peak around 1380 cm^−1^ indicates the N-O stretching vibration [50] of NO_3_ ^−^, which remains present until 500 °C. The peak situated at 1530 cm^−1^ can be attributed to the asymmetrical stretching of C-O, while the absorption bands at 1090 cm^−1^ and 850 cm^−1^ are due to the symmetrical stretching of C-O and deformation vibrations of C-O in CO_3_ ^2−^. These absorption peaks indicate the presence of carbonate groups. Bands of C-O-H and CH_2_- corresponding to bending vibrations appear around 1410 cm^−1^ and 1460 cm^−1^, respectively, arising from the decomposition of DEG [51], and one is observed at 820–880 cm^−1^, characteristic of the C-C bond [52]. The intensity of all these bands decreased with the annealing temperature, as has been observed for lutetia ceramics fabricated by nitrate sources [53]. However, after 800 °C sintering, the N-O vibrations were still present, suggesting that some NO_3_ ^−^ residues were adsorbed in the sample, which could be removed after 900 °C thermal treatment. Finally, the bands at 580 and 489 cm^−1^, observed from 400 °C, and attributed to the Lu-O stretching vibrations of cubic Lu_2_O_3_ (a Lu_2_O_3_ host lattice vibration) [54–56], indicate that the crystallization was just beginning at 400 °C, which was confirmed by XRD and TEM observations.

### 2.3. Structural Properties

**Figure 3:**
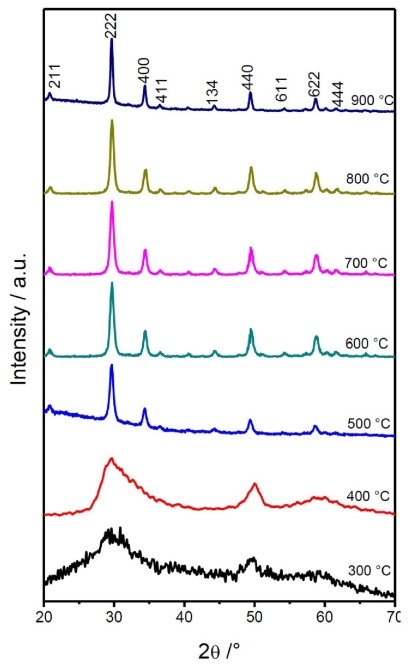
Structural evolution of Lu_2_O_3_: 5 mol % Eu^3+^, 0.01 mol % Tb^3+^ powders as function of thermal treatment.

Figure 3 shows the evolution of the X-ray diffraction patterns of the Lu_2_O_3_: Eu^3+^ 5 mol %, Tb^3+^ 0.01 mol% nanopowders annealed in air at temperatures ranging from 300 to 900 °C. At 300 °C, the xerogel exhibits an almost amorphous behavior; however, at 400 °C, the system possesses an aspect of an amorphous phase characterized by broad diffraction peaks, which also indicates that the crystallites are very small, lower than 5 nm.

This result is in good agreement with the DTA observations, since the crystallization process began between 300 and 400 °C. As the annealing temperature reaches 500 °C, it becomes evident that the crystallization process has ended in a cubic Lu_2_O_3_ structure (JCPDS 431021) with a spatial group Iā3 (lattice parameter 10.391 Å). With an increments the annealing temperature, the diffraction peaks become narrower, which reflects an increase in the size of the Lu_2_O_3_ crystallites. Table 1 shows the calculated crystal sizes according to Scherer’s formula D = 0.9λ/β cosθ [57], taking into account the broadening line of the diffracted peak, due to the effect of crystal size, where D is the crystal size of the powder, λ (0.15406 nm) is the wavelength of the diffracted X-ray, *β* is the full-width radiation at half-maximum (FWHM) of the peak, and θ is the Bragg angle of the diffracted X-ray. The crystallite size ranges from 5.3 nm at 500 °C to 25.5 nm at 900 °C; these observations were confirmed by TEM observations for the 700 and 900 °C co-doped lutetia nanopowders.

**Figure 4:**
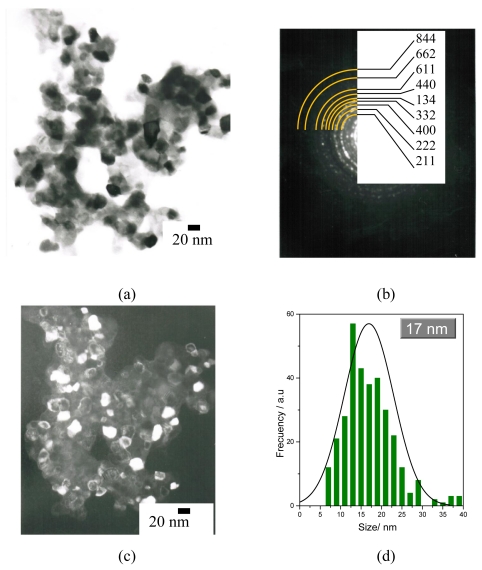
(**a**) TEM bright field micrograph of Lu_2_O_3_ 5 mol %Eu^3+^, 0.01 mol % Tb^3+^ powders annealed at 700 °C; (**b**) XRD diffraction pattern; (**c**) TEM dark field at (222) plane; (**d**) crystal size distribution.

Figure 4a shows a TEM bright-field micrograph of selected Lu_2_O_3_: Eu^3+^ 5 mol %, Tb^3+^ 0.01 mol % scintillating nanopowders, annealed at 700 °C. As observed, the morphology of the powders is mainly angular; however, some of the faces are rounded and the particles are highly agglomerated. Figure 4b shows the particles’ indexed diffraction pattern, which exhibits typical nanometric ring-type behavior and confirms the cubic structure. Figure 4c shows a dark field micrograph of the area at the (2 2 2) direction. The average size determined from these observations was ≈ 17 nm, in good accord with XRD results, and presents a normal centered distribution in the range of 5–40 nm (Figure 4d).

**Figure 5:**
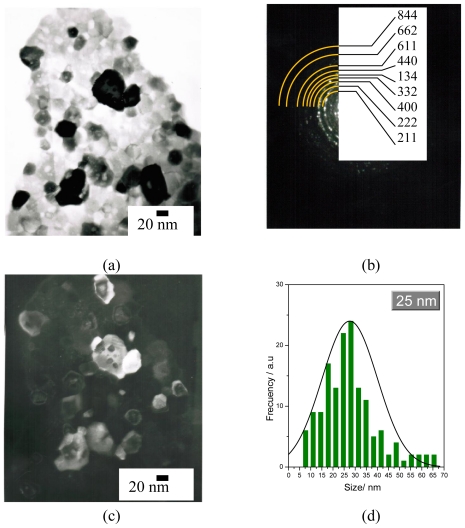
(**a**) TEM bright field micrograph of Lu_2_O_3_ 5 mol%Eu^3+^, 0.01 mol % Tb^3+^ powders annealed at 900 °C; (**b**) XRD diffraction pattern; (**c**) TEM dark field at (222) plane; (**d**) crystal size distribution.

Bright field, diffraction pattern and dark field micrographs, as well as particle size distribution of the sample annealed at 900 °C are shown in Figure 5a–b. As noted, similar observations can be made for this sample, whose average size was ≈ 27 nm. The kinetics growing of the sample can be related to the sintering and agglomeration processes activated by the thermal process.

### 2.4. Scintillating Properties

**Figure 6:**
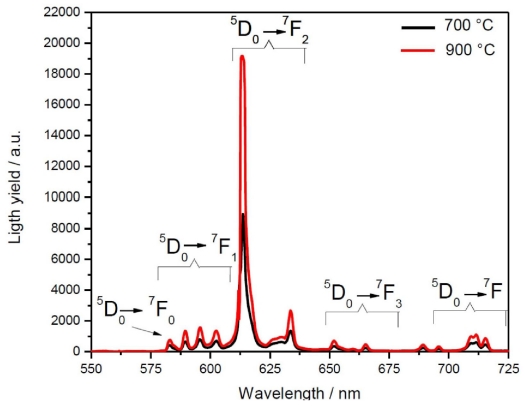
Emission spectra of Lu_2_O_3_: 5 mol % Eu^3+^, 0.01 mol % Tb^3+^ powders annealed at 700 and 900 °C under X-ray excitation.

Figure 6 presents the scintillating properties of Lu_2_O_3_: Eu^3+^ 5 mol%, Tb^3+^ 0.01 mol% nanopowders annealed at 700 and 900 °C, under X-ray excitation of 30 kV and 40 mA. The scintillating spectra of the sample display a group of emission lines situated in the 575- to 725-nm spectral region, corresponding the Eu^3+^ transitions from the excited ^5^D_0_ level to ^7^F_j_ (J = 0,1,2,3,4) levels, with no evidence of any Tb^3+^ emission (545 nm). The maximum emission line at 611 nm, which matches well the spectral sensitivity range of CCD cameras, corresponds to the ^5^D_0_ → ^7^F_2_ electric dipole transition, and is a product of the Eu^3+^ ion located at C_2_ sites in the Lu_2_O_3_ host cubic lattice [58], and its intensity is evidence of an efficient channel of energy transfer from the lutetia matrix to Eu^3+^ emission centers according to a recombination mechanism [59]. The less intense emission lines at 532, 580, 630 and 665 nm, correspond to the ^5^D_0_→^7^F_0_, ^5^D_0_→^7^F_1_, ^5^D_0_→^7^F_3_ and ^5^D_0_→^7^F_4_ transitions, respectively, and are in good agreement with the energy levels described elsewhere [60]. The difference in the light yield output between the two samples, of about ~110%, can be explained in terms of two effects. First, it is well known that the crystallinity achieved at higher temperatures is related to a more efficient activation of the Eu^3+^ ion in the nanopowder sample. Furthermore, it has been demonstrated that until the complete elimination of residual OH contamination, the non-radiative multiphonon relaxation process can be stimulated with high-energy phonons introduced by this impurity [33]. In the current work, as can be established by IR experiments, at 900 °C the residual OH^−^ impurity has been almost completely eliminated, which is a temperature considerably lower to the 1300 °C range that has been previously reported [61].

**Figure 7:**
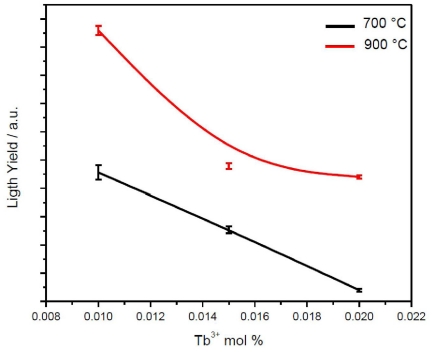
Evolution of the scintillating light yield of Lu_2_O_3_: (5 mol % Eu^3+^, X mol % Tb^3+^ powders annealed at 700 and 900 °C as a function of Tb^3+^.

Figure 7 shows the light yield variation of the Eu^3+ 5^D_0_→^7^F_2_ (611 nm) emission as a function of the Tb^3+^ concentration for nanopowders annealed at 700 and 900 °C. In both cases, the lutetia co-doped at 0.01 mol % Tb^3+^ presents the highest light yield, and for reasons explained earlier, the emission of the sample heat-treated at 900 °C is higher than that of the 700 °C one. The enhancement of the Eu^3+^ emission in the nanopowders is due to a non-radiative energy transfer from Tb^3+^ to Eu^3+^, as Tb can absorb more X-ray radiation. At higher Tb^3+^ concentrations, a self-quenching mechanism [45] exists, a product of the fact that the Tb-Tb energy transfer mechanism is more efficient than the Tb-Eu one, and, therefore, presents a higher possibility that a Tb ion is closer than another sort, resulting in a drop in Eu^3+^ emission. Similar results have been observed for Gd_2_O_3_ co-doped Eu, Tb nanopowders [47].

## 3. Experimental Section

**Figure 8:**
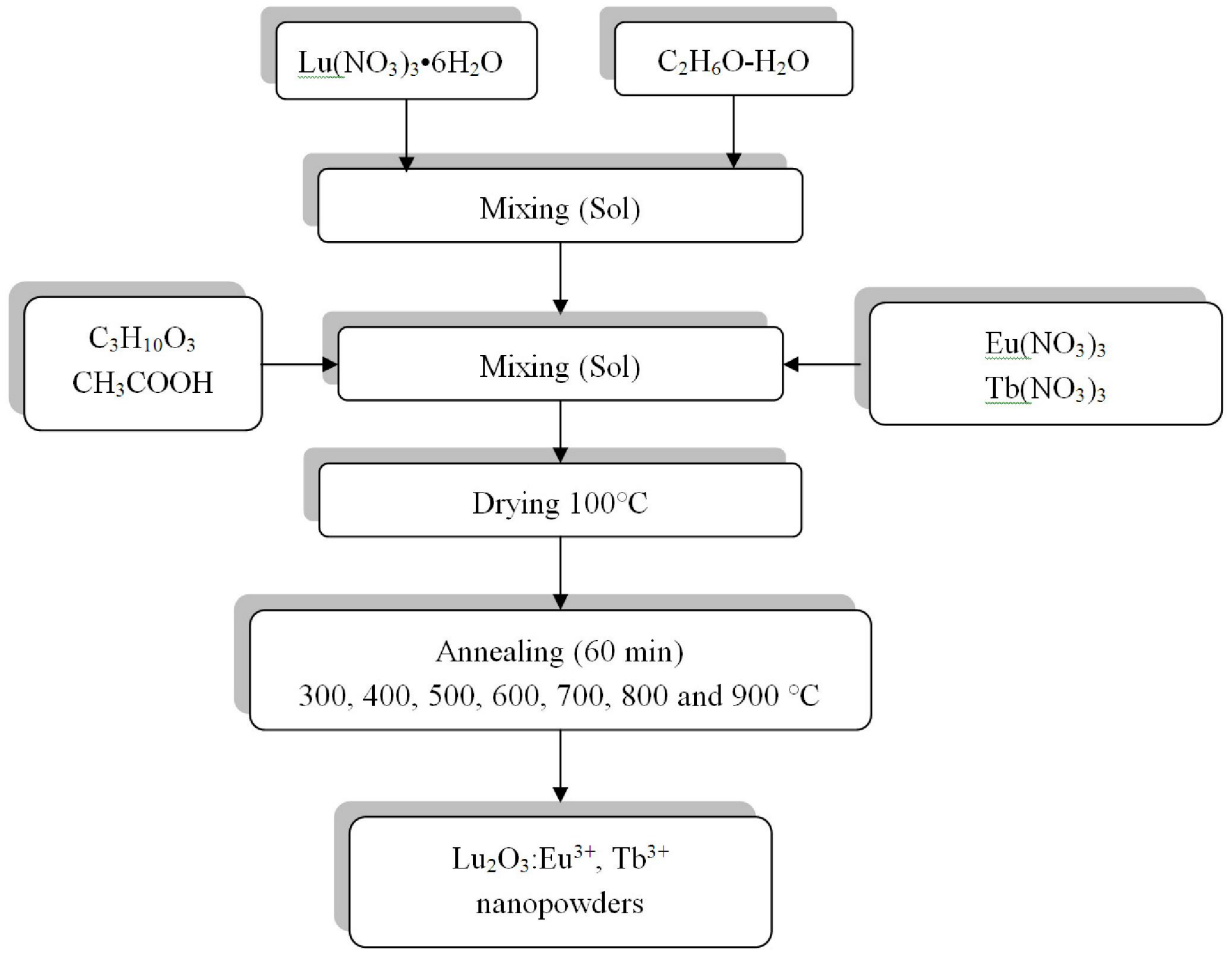
Sol-gel process of Lu_2_O_3_:Eu^3+^, Tb^3+^ nanopowders synthesis scheme.

Lu_2_O_3_:Eu^3+^, Tb^3+^ nanopowders were prepared by a simple sol-gel process method. Figure 8 shows the flow scheme for this process. The starting materials used were lutetium nitrate, Lu(NO_3_)_3_•6H_2_O (Alfa Aesar, 99.96%), europium nitrate Eu(NO_3_)_3_, terbium nitrate Tb(NO_3_)_3_ (99.5% and 99.6% respectively, Alfa Aesar), acetic acid CH_3_COOH (Fermont 98%) used as a catalyst, and diethyleneglycol (DEG) C_4_H_10_O_3_ (Alfa Aesar, 99%) used as a polymerization agent. Initially, the lutetium nitrate was dissolved in an ethanol-deionized water solution (95-5 vol%) using a hot stirrer at 40 °C for 3 hours to obtain 25 mL of a 0.95 M Lu sol. Thereafter, the solution pH was adjusted by incorporating acetic acid (0.17 M), and the DEG was added (0.42 M). Finally, the co-doping elements, previously dissolved in ethanol, were incorporated the initial lutetium sol. The sol obtained was mixed for 2 hours to produce a transparent solution, stable for more than 3 months. Subsequently, the sol was dried at 100 °C for 24 hours, and the yielded gel was calcined at different temperatures for 1 hour to obtain the Lu_2_O_3_:Eu^3+^, Tb^3+^ scintillating nanopowders.

Thermogravimetric (TGA) and differential thermal analyses (DTA) of dried gel were conducted using a SDT Q600 TA instrument; the studies were performed in air with a heating rate of 10 °C min^−1^. The IR spectra of the samples were recorded in the range of 4000–400 cm^−1^ using Fourier transform infrared spectroscopy (FTIR 2000, Perkin Elmer) and the KBr pelleting technique. The phase composition of the powders was identified by X-ray diffraction at room temperature on a powder diffractometer (Bruker D8Advance) using Cu Kα radiation (1.5418 Å). The morphology and particle size of the powders were observed with a transmission electronic microscope (JEOL 2200) operating at 200 keV. The scintillating properties were recorded employing an X-ray generator (PW-1830 Phillips) operating at 30 kV and 40 mA, using W Kα radiation (0.2086 Å) and a photon flux of 10^12^ ph s^−1^.

## 4. Conclusions

Lu_2_O_3_:Eu^3+^, Tb^3+^ nanopowders have been prepared by a simple sol-gel method, using lutetium, europium and terbium nitrate as precursors, and DEG as a polymerization agent. This nanopowder crystallizes into a cubic system from 400 °C, and the process is completed at 500 °C, resulting in particles composed of crystallites ranging in size from 5.3 to 25.5 nm. The increments in crystallite sizes depend on the heat treatment temperatures. The powder exhibits interesting scintillating properties, and at 611 nm presents a reddish emission corresponding with the Eu^3+ 5^D_0_→^7^F_2_ transition, which makes it a promising material for X-ray detection systems, since this emission matches the maximum efficiency of the CCD cameras. The light yield of the nanopowders was analyzed at two different annealing temperatures: 700 and 900 °C. It was established that at the sample heat treated at the higher temperature, presents an enhanced light output, presumably due to better crystallinity and to the complete removal of OH^−^. Finally, it was determined that with the co-doping of Tb^3+^ at a 0.01 mol% level, the light yield was enhanced compared to trials with higher Tb contents, presumably due to the effect of a self-quenching process.

## Figures and Tables

**Table 1:** Crystallite size as function of annealing temperature.

Temperature/°C	500	600	700	800	900
FWHM/Degree	1.94	0.75	0.57	0.49	0.38
Crystal Size/nm	5	14	18	19	26

## References

[1] Zych E, Hreniak D, Stark W (2002). Lu_2_O_3_:Eu, a new X-ray phosphor. Mater Sci.

[2] Van Eijik CWE (2001). Inorganic-scintillator development. Nucl Intrum Meth A.

[3] Greskovich C, Duclos S (1997). Ceramic scintillators. Annu Rev Mater Sci.

[4] Zych E, Meijerink A, Mello Donega C (2003). Quantum efficiency of europium emission from nanocrystalline powders of Lu_2_O_3_:Eu. J Phys Condens Mat.

[5] Liaparinos PF, Kandarakis IS (2009). The imaging performance of compact Lu_2_O_3_:Eu powdered phosphor screens: Monte Carlo simulation for applications in mammography. Med Phys.

[6] Zych E, Halwa HS, Rohwer LS (2003). Lumininscence and scintillation of inorganic phosphor materials. Handbook of Luminiscence Dysplay Materials and Devices.

[7] Garcia-Murillo A, Le Luyer C, Dujardin C, Martin T, Garapon C, Pedrini C (2002). Elaboration and scintillation properties of Eu^3+^-doped Gd_2_O_3_ and Lu_2_O_3_ sol-gel films. Nucl Instrum Meth A.

[8] Zych E, Trojan-Piegza J, Dorenbos P (2004). Radioluminescence of Lu_2_O_3_:Eu nanocrystalline powder and vacuum-sintered ceramic. Radiat Meas.

[9] Nagarkar VV, Miller SR, Tipnis SV, Lempikchi A, Brecher C, Lingertat H (2004). A new large area scintillator screen for X-ray imaging. Nucl Instrum Meth B.

[10] Zych E, Hreniak D, Strek W (2002). Spectroscopic properties of Lu_2_O_3_/Eu^3+^ nanocrystalline powders and sintered ceramics. J Phys Chem B.

[11] Liu XJ, Lio HL, Xie RJ, Hirosaki N, Xu X, Huang LP (2007). Synthesis, characterization, and luminescent properties of Lu_2_O_3_:Eu phosphors. J Lumin.

[12] Lempicky A, Brecher C, Szupryczynski P, Lingertat H, Nagarkar VV, Tipnis SV, Miller SR (2002). A new lutetia-based ceramic scintillator for X-ray imaging. Nucl Instrum Meth A.

[13] Quaranta A, Gramegna F, Kravchuk V, Scian C (2008). Radiation damage mechanism in CsI Tl studied by ion beam induced luminiscence. Nucl Instrum Meth B.

[14] Nik M, Yoshikawa A, Vedda A, Fakuda T (2006). Development of novel scintillator crystals. J Cryst Growth.

[15] Lalic MV, Souza SO (2008). The fist principles study of electronic and optical properties of BGO and BSO scintillators. Opt Mater.

[16] Sthephen G, Topping V, Sarin K (2009). CVD Lu_2_O_3_:Eu^3+^ coatings for advanced scintillators. Int J Refract Metals Hard Mater.

[17] Jones SL, Kumar D, Sing PK, Hollouway PH (1997). Luminescence of pulsed laser deposited Eu doped yttrium oxide films. Appl Phys Lett.

[18] Kumar D, Sankar J, Cho KG, Cracium V, Singh RK (2000). Enhancement of cathodoluminescent and photoluminescent properties of Eu:Y_2_O_3_ luminescent films by vacuum cooling. Appl Phys Lett.

[19] Cho S, Lee H, Moon C, Kim J, Park J, Jeon G, Lee R, Nam S (2010). Synthesis and characterization of Eu^3+^ doped Lu_2_O_3_ nanophosphor using a solution-combustion method. J Sol-Gel Sci Tech.

[20] Farman TT, Gakenheimer DC, Lempicki A, Miller SR, Scheetz JP, Shafie A, Farman AG (2003). Computer-aided maxillofacial radiographic diagnosis: Impact of variations in scintillator and acquisition mode. Int Congr Ser.

[21] Antic-Findacev E, Hölsä J, Lastusaari M (2003). Crystal field energy levels of Eu^3+^ and Yb^3+^ in the C_2_ and S_6_ sites of the cubic C-type R_2_O_3_. J Phys Condes Matter.

[22] Daldosso M, Sokolnicki J, Kepinski L, Legendziewicz J, Speghini A, Bettinelli M (2007). Preparation and optical properties of nanocrystalline Lu_2_O_3_:Eu^3+^ phosphors. J Lumin.

[23] Wang Z, Zhang W, Lin L, You B, Fu Y, Yin M (2008). Preparation and spectroscopic characterization of Lu_2_O_3_:Eu^3+^ nanopowders and ceramics. Opt Mater.

[24] Jia G, You H, Zheng Y, Liu K, Guo N, Zhang H (2010). Synthesis and characterization of highly uniform Lu_2_O_3_:Ln^3+^ (Ln = Eu, Er, Yb) luminescent hollow microspheres. Cryst Eng Comm.

[25] Lu Z, Chen L, Tang Y, Li Y (2005). Facile synthesis and characterization of sheet-like Y_2_O_3_:Eu^3+^ microcrystals. J Cryst Growth.

[26] Dulina NA, Yermolayeva YV, Tolmachev AV, Sergienko ZP, Vovk OM, Vovk EA, Matveevskaya NA, Mateychenko PV (2010). Synthesis and characterization of the crystalline powders on the basis of Lu_2_O_3_:Eu^3+^ spherical submicron-sized particles. J Eur Ceram Soc.

[27] Yin S, Akita S, Shinozaki M, Li R, Sato T (2008). Synthesis and morphological control of rare earth oxide nanoparticles by solvothermal reaction. J Mater Sci.

[28] Li Y, Zhang J, Luo Y, Zhang X, Hao Z, Wang X (2011). Color control and white light generation of upconversion luminescence by operating dopant concentrations and pump densities in Yb^3+^, Er^3+^ and Tm^3+^ tri-doped Lu_2_O_3_ nanocrystals. J Mater Chem.

[29] Qiu HJ, Jun QH, Xie JJ, Ji X, Lin X, Xu FF (2010). Hydrothermal route to Eu doped LuO(OH) and Lu_2_O_3_ nanorods. Sci Chiba Tech Sci.

[30] Wang J, Liu Q, Liu Q (2007). Synthesis and luminescence properties of Eu or Tb doped Lu_2_O_3_ square nanosheets. Opt Mater.

[31] Li L, Yang HK, Moon BK, Choi BCh, Jeong JH, Kim KH (2010). Photoluminescent properties of Ln_2_O_3_:Eu^3+^ (Ln = Y, Lu and Gd) prepared by hydrothermal process and sol-gel method. Mat Chem Phys.

[32] Trojan-Piegza J, Zych E (2004). Preparation of nanocrystalline Lu_2_O_3_:Eu phosphor via a molten salts route. J Alloy Compd.

[33] Zych E, Trojan-Piegza J, Kepinsky L (2005). Homogeneously precipitated Lu_2_O_3_:Eu nanocrystalline phosphor for X-ray detection. Sensor Actuat B Chem.

[34] Chen QW, Shia Y, Chena JY, Shia JL (2005). Photoluminescence of Lu_2_O_3_:Eu^3+^ phosphors obtained by glycine-nitrate combustion synthesis. J Mater Res.

[35] Qi Z, Liu M, Chen Y, Zhang G, Xu M, Shi C, Zhang W, Yin M, Xie Y (2007). Local structure of nanocrystalline Lu_2_O_3_:Eu studied by X-ray absorption spectroscopy. J Phys Chem C.

[36] William Barrera E, Cinta Pujol M, Cascales C, Carvajal JJ, Mateos X, Aguiló M, Diaz F (2011). Synthesis and structural characterization of Tm:Lu_2_O_3_ nanocrystals. An approach towards new laser ceramics. Opt Mat.

[37] Sokolnicky J (2007). Photoluminescence and structural characteristics of Lu_2_O_3_:Eu^3+^ nanocrystallites in silica matrix. J Solid State Chem.

[38] Chen Q, Shi Y, An L, Wang S, Chen J, Shi J (2007). A novel co-precipitation synthesis of a new phosphor Lu_2_O_3_:Eu^3+^. J Eur Ceram Soc.

[39] Nedelec JM (2007). Sol-gel processing of nanostructured inorganic scintillating materials. J Nanomater.

[40] Hreniak J, Zych E, Kepinsky L, Strek W (2003). Structural and spectroscopic studies of Lu_2_O_3_/Eu^3+^ nanocrystallites embedded in SiO_2_ sol-gel ceramics. J Phys Chem Solids.

[41] Yan J, Li J (2010). Sol-gel synthesis of nanocrystalline Yb^3+^/Ho^3+^-doped Lu_2_O_3_ as an efficient green phosphor. J Electrochem Soc.

[42] García-Murillo A, Carrillo-Romo FJ, Le Luyer C, Morales-Ramírez AJ, García-Hernández M, Moreno-Palmerin J (2009). Sol-gel elaboration and structural investigations of Lu_2_O_3_ planar waveguides. J Sol-Gel Sci Tech.

[43] Guo H, Yin M, Dong N, Xu M, Lou L, Zhang W (2005). Effect of heat-treatment temperature on the luminescent properties of Lu_2_O_3_:Eu film prepared by Pechini sol-gel method. Appl Surf Sci.

[44] Liu Y, Yang Y, Qian G, Wang Z, Wang M (2007). Energy transfer processes from Tb^3+^ to Eu^3+^ in ternary chelate doped in gel glasses via *in situ* technique. Mat Sci Eng B.

[45] Mukherjee S, Sudarsan V, Vatsa RK, Godbole SV, Kadam RM, Bhatta UM, Tyagi AK (2008). Effect of structure, particle size and relative concentration of Eu^3+^ and Tb^3+^ ions on the luminescence properties of Eu^3+^ co-doped Y_2_O_3_:Tb nanoparticles. Nanotechnology.

[46] Liu Z, Yu L, Wang Q, Tao Y, Yang H (2011). Effect of Eu,Tb codoping on the luminescent properties of Y_2_O_3_ nanorods. J Lumin.

[47] Morales-Ramírez A (2010). de J., García-Murillo, A., Carrillo-Romo, F. de J., García-Hernández, M., Jaramillo-Vigueras, D., Chaderyron G., Boyer, D. Properties of Gd_2_O_3_:Eu^3+^, Tb^3+^ nanopowders obtained by sol-gel process. Mater Res Bull.

[48] Chen Q, Ying S, An L, Wang S, Chen J, Shi J (2007). A novel co-precipitation synthesis of a new phosphor Lu_2_O_3_:Eu^3+^. J Eur Ceram Soc.

[49] Zych E (2001). On the reasons for low luminescence efficiency in combustion-made Lu_2_O_3_:Tb. Opt Mater.

[50] Chi Y, Chuang S (2000). Infrared and TPD studies of nitrates adsorbed on Tb_4_O_7_, La_2_O_3_, BaO, and MgO/γ-Al_2_O_3_. J Phys Chem B.

[51] Stefanescu M, Stoia M, Stefanescu O (2007). Thermal and FT-IR study of the hybrid ethylene-glycol-silica matrix. J Sol-Gel Sci Tech.

[52] Ksapabutr B, Gulari E, Wongkasemjit S (2004). One-pot synthesis and characterization of novel sodium tris(glycozirconate) and cerium glycolate precursors and their pyrolisis. Mater Chem Phys.

[53] Shi Y, Chen QW, Shi JL (2009). Processing and scintillation properties of Eu^3+^ doped Lu_2_O_3_ transparent ceramics. Opt Mater.

[54] Socrates G (2001). Infrared Characteristic Grooup Frequencies. Table and Charts.

[55] McDevitt NT, Baun WL (1964). Infrared absorption study of metal oxides in the low frequency region (700–240 cm^−1^). Spectrochim Acta.

[56] Garcia-Murillo A, Le Luyer C, Pedrini C, Mugnier J (2001). Synthesis and properties of Lu_2_O_3_ sol-gel films. J Alloys Compd.

[57] Cullity BD (1978). Elements of X-Ray Diffraction.

[58] Zych E (2002). Concentration dependence of energy transfer between Eu^3+^ ions occupying two symmetry sites in Lu_2_O_3_. J Phys Condens Mater.

[59] Zych E, Trojan-Piegza J (2006). Low-temperature luminescence of Lu_2_O_3_:Eu ceramics upon excitation with synchrotron radiation in the vicinity of band gap energy. Chem Mater.

[60] Karbowiak M, Zych E, Holsa J (2003). Crystal-field analysis of Eu^3+^ in Lu_2_O_3_. J Phys Condens Matter.

[61] Zych E, Deren PJ, Strek W, Meijerink A, Mielcarek W, Dmagala K (2001). Preparation, X-ray analysis and spectroscopic investigation of nanostructured Lu_2_O_3_:Tb. J Alloy Compd.

